# A Rapid, Whole Genome Sequencing Assay for Detection and Characterization of Novel Coronavirus (SARS-CoV-2) Clinical Specimens Using Nanopore Sequencing

**DOI:** 10.3389/fmicb.2022.910955

**Published:** 2022-06-06

**Authors:** Maria T. Arévalo, Mark A. Karavis, Sarah E. Katoski, Jacquelyn V. Harris, Jessica M. Hill, Samir V. Deshpande, Pierce A. Roth, Alvin T. Liem, R. Cory Bernhards

**Affiliations:** ^1^Defense Threat Reduction Agency, Aberdeen Proving Ground, MD, United States; ^2^United States Army Combat Capabilities Development Command Chemical Biological Center, Aberdeen Proving Ground, MD, United States; ^3^DCS Corporation, Belcamp, MD, United States

**Keywords:** nanopore sequencing, COVID-19, SARS-CoV-2, whole genome sequencing, whole genome assembly

## Abstract

A new human coronavirus, severe acute respiratory syndrome coronavirus 2 (SARS-CoV-2), emerged at the end of 2019 in Wuhan, China that caused a range of disease severities; including fever, shortness of breath, and coughing. This disease, now known as coronavirus disease 2019 (COVID-19), quickly spread throughout the world, and was declared a pandemic by the World Health Organization in March of 2020. As the disease continues to spread, providing rapid characterization has proven crucial to better inform the design and execution of control measures, such as decontamination methods, diagnostic tests, antiviral drugs, and prophylactic vaccines for long-term control. Our work at the United States Army’s Combat Capabilities Development Command Chemical Biological Center (DEVCOM CBC) is focused on engineering workflows to efficiently identify, characterize, and evaluate the threat level of any potential biological threat in the field and more remote, lower resource settings, such as forward operating bases. While we have successfully established untargeted sequencing approaches for detection of pathogens for rapid identification, our current work entails a more in-depth sequencing analysis for use in evolutionary monitoring. We are developing and validating a SARS-CoV-2 nanopore sequencing assay, based on the ARTIC protocol. The standard ARTIC, Illumina, and nanopore sequencing protocols for SARS-CoV-2 are elaborate and time consuming. The new protocol integrates Oxford Nanopore Technology’s Rapid Sequencing Kit following targeted RT-PCR of RNA extracted from human clinical specimens. This approach decreases sample manipulations and preparation times. Our current bioinformatics pipeline utilizes Centrifuge as the classifier for quick identification of SARS-CoV-2 and RAMPART software for verification and mapping of reads to the full SARS-CoV-2 genome. ARTIC rapid sequencing results, of previous RT-PCR confirmed patient samples, showed that the modified protocol produces high quality data, with up to 98.9% genome coverage at >1,000x depth for samples with presumably higher viral loads. Furthermore, whole genome assembly and subsequent mutational analysis of six of these sequences identified existing and unique mutations to this cluster, including three in the Spike protein: V308L, P521R, and D614G. This work suggests that an accessible, portable, and relatively fast sample-to-sequence process to characterize viral outbreaks is feasible and effective.

## Introduction

A new coronavirus, severe acute respiratory syndrome coronavirus 2 (SARS-CoV-2) emerged in Wuhan, China in 2019. It was quickly sequenced and identified as being related to severe acute respiratory syndrome (SARS) virus, with some homology to bat coronaviruses (Andersen et al., 2020; Lu et al., 2020; Wang et al., 2020; Wu et al., 2020). The disease caused by this novel coronavirus, COVID-19, was discovered in a cluster of pneumonia cases associated with a Huanan seafood market. At the time, the most common symptoms reported at the onset of illness were fever, cough, myalgia, and fatigue (Huang et al., 2020). The hospitalized patients all had pneumonia with acute respiratory distress syndrome (ARDS) as a common complication and a high fatality rate of 15% (Huang et al., 2020). Since then, the virus and disease have spread, causing a pandemic that has yet to be controlled.

SARS-CoV-2 has a single-stranded, positive sense RNA genome with a 5′ cap and poly A tail (Romano et al., 2020). Its RNA genome has 14 open-reading frames (ORFS) that encode 16 non-structural proteins (Nsp1-16) that are involved in replication, and 4 structural proteins (spike—S, envelope—E, membrane—M, and nucleocapsid—N) that are assembled into the virion (Romano et al., 2020). When it comes to structural proteins, the trimeric S protein is particularly important because it mediates host cell receptor binding and entry. The S protein is also a main target of the neutralizing antibody response and thus, the majority of developing vaccine and antibody-based therapeutic approaches are directed against it (Korber et al., 2020). Monitoring changes in this protein will be particularly important because mutations in this protein may alter the phenotype of the virus, transmission, and effect efficacy of vaccines and other medical countermeasures that have been developed using strains that were identified and isolated in Wuhan, China early on in the pandemic.

Clinical isolates from around the globe have been sequenced, shared and published in databases such as GenBank and GISAID (Shu and McCauley, 2017). GISAID as an example, has received over 10 million genome sequence submissions as of April 12, 2022^1^. Furthermore, GISAID has introduced a nomenclature system for major clades (GISAID, 2020). Classification is based on marker mutations from the early split of clades S and L, evolution of L into V and G, and then G into GH, GR, and then GV. The current GISAID clades (GISAID, 2020) are shown in Table 1, with comparison to other classification schema, and including variants of concern as designated by the WHO (WHO, 2022).

**TABLE 1:** SARS-CoV-2 GISAID clade classifications, corresponding pango lineage, and variants of concern.

GISAID clade	Pango lineage	Marker variants	Variants of concern
S	A	C8782T, T28144C includes NS8-L84S	
L	B	C241, C3037, A23403, C8782, G11083, G26144, T28144 (early clade markers in WIV04- GISAID reference sequence)	
V	B.2	G11083T, G26144T NSP6-L37F + NS3-G251V	
G	B.1	C241T, C3037T, A23403G includes S-D614G	
GH	B.1.*	C241T, C3037T, A23403G, G25563T includes S-D614G + NS3-Q57H	Beta (B.1.3151)
GR	B.1.1.1	C241T, C3037T, A23403G, G28882A includes S-D614G + N-G204R	Gamma (P.1 or B.1.1.28.1)
GV	B.1.177	C241T, C3037T, A23403G, C22227T includes S-D614G + S-A222V	
GRY	B.1.1.7	C241T, C3037T, 21765-21770del, 21991-21993del, A23063T, A23403G, G28882A includes S-H69del, S-V70del, S-Y144del, S-N501Y + S-D614G + N-G204R	Alpha
GK	B.1.617.2	C241T, C3037T, A23403G, C22995A S-D614G + S-T478K	Delta
GRA	B.1.1.529	A67V, del69-70, T95I, del142-144, Y145D, del211, L212I, ins214EPE, G339D, S371L, S373P, S375F, K417N, N440K, G446S, S477N, T478K, E484A, Q493R, G496S, Q498R, N501Y, Y505H, T547K, D614G, H655Y, N679K, P681H, N764K, D796Y, N856K, Q954H, N969K, L981F	Omicron (includes BA.1-BA.5 or B.1.1.529.1-B.1.1.529.5, XE-recombinant BA.1/BA.2)

The majority of whole genome sequencing for molecular epidemiology employ Illumina next-generation sequencing technology (Seth-Smith et al., 2019); this platform is currently considered the gold-standard for data quality and accuracy. However, Illumina equipment has a large footprint in terms of space, power consumption and requires a certified technician for set up and maintenance, making the technology less operable in the field and less obtainable in remote regions of the world where new pathogens of interest may emerge. Oxford Nanopore Technology’s (ONT, Oxford, United Kingdom) MinION sequencers, which rely on use of nanopores for sequencing, are hand-held portable devices that are accessible and easy to set up anywhere, without an ONT technician. Thus, the MinION handheld sequencers are an attractive alternative technology for rapid and fieldable deployment. Moreover, the ARTIC Network has been developing end-to-end protocols utilizing this technology to sequence RNA viruses that include Ebola, influenza, and more recently SARS-CoV-2. The original ARTIC SARS-CoV2 protocol was released in early January 2020, enabling sequencing in different countries of the world, early on in the pandemic (Tyson et al., 2020). It has since become a widely used approach and more recently, a head-on comparison of ARTIC sequencing assays with Illumina versus nanopore sequencing showed these yielded similar results with respect to coverage and identification of variants (Charre et al., 2020).

The ARTIC Network’s SARS-CoV-2 protocol for nanopore sequencing relies on direct amplification of the reverse-transcribed viral genome using a tiled, multiplexed, primer approach. The primer scheme is based on GenBank accession MN908947, released shortly after identification of the virus (Artic-Network, 2020). The protocol is highly sensitive, making it possible to sequence viruses directly from clinical samples. The protocol has also been adopted by investigators worldwide; primer sets have been published and are available commercially as a full set. However, while effective and highly sensitive, the ARTIC SARS-CoV-2 protocol is elaborate and time-consuming. In this study, we describe a modified ARTIC process that decreases sample manipulation and preparation times, resulting in high quality data that can be used downstream for viral genome assembly and analyses. The new protocol was evaluated using a panel of CoV-2 positive and negative clinical samples as previously diagnosed by RT-PCR assays.

## Materials and Methods

### Cohort

Twenty positive samples and twenty negative samples were received from Justin T. Bacca’s group at the University of New Mexico. The samples were tested by their reference lab *via* EUA cleared RT-PCR assays and were provided as TRIzol-inactivated samples (2 parts Trizol to 1 part sample in VTM).

### RNA Extractions

The total RNA was extracted from the TRIzol-inactivated clinical samples by using the Direct-zol RNA MicroPrep Kit (catalog number R2060) from Zymo Research. The manufacturer’s instructions were followed with the exception of the elution volume being doubled from a volume of 15 to 30 μL. After the RNA was extracted, the concentration and quality of the RNA was determined by Nanodrop analysis.

### Library Preparations

**FIGURE 1:**
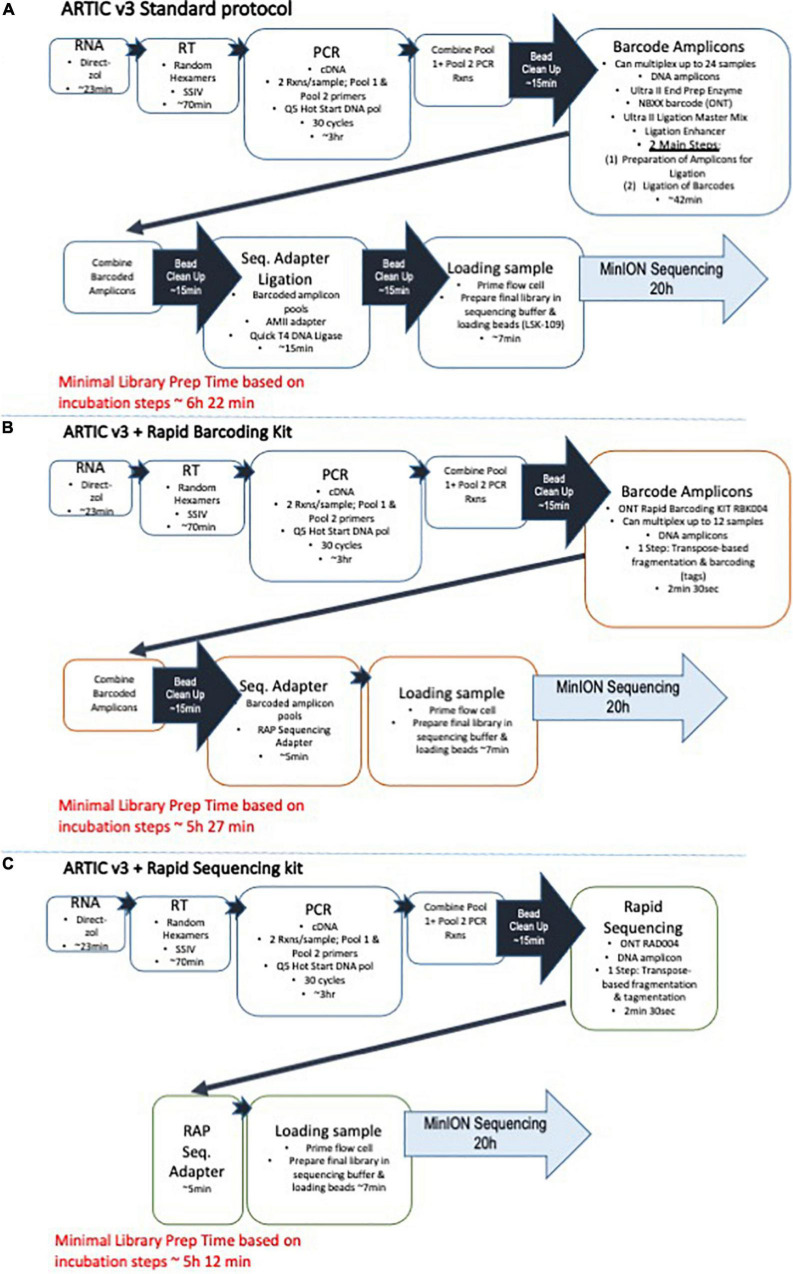
Artic protocol and modified versions. Schematics for the COVID-19 sequencing workflows are shown starting with **(A)** the standard Artic protocol with v3 primers and followed by **(B)** the Artic protocol modified for use with the Rapid Barcoding Sequencing kit for multiplexed samples, and **(C)** the Artic protocol modified for use with the Rapid Sequencing kit for rapid sequencing of individual samples.

The RNA was converted to cDNA and amplified using a targeted approach developed by the ARTIC Network. The ARTIC “nCoV-2019 sequencing protocol” (Quick, 2020) was followed precisely, except for a couple steps as described and can be visualized in Figure 1A. The Artic’s V3 primer panel (Tyson et al., 2020), consisting of two pools of primer pairs with 98 primers in each pool were used in the amplification of the cDNA (ARTIC nCoV-2019 V3 Panel, 500rxn, Catalog number 10006788) from Integrated DNA Technologies (Coralville, IA, United States). Two PCR reactions per sample were prepared, one using the first set of the primer pool (primer pool #1) and another using the second primer set (primer pool #2). During the PCR amplification step, 10 μL of cDNA was used instead of 2.5 μL for each reaction to maximize the amount of product going into the amplification. Thirty cycles of amplification was performed using the Q5 Hot Start High Fidelity DNA Polymerase (M0493L, New England Biolabs/NEB, Ipswich, MA, United States) and Applied Biosystems GeneAmp 9700 thermocycler. After amplification, the two ARTIC PCR reactions per sample are combined together, and this is followed by an AMPure XP (A63880, Beckman-Coulter, Indianapolis, IN, United States) bead DNA clean-up step. The concentration of the eluted DNA was determined by Qubit analysis with the Qubit dsDNA HS Assay Kit (Q33231, Thermo Fisher Scientific, Waltham, MA, United States). Using the standard protocol, the amplicons were prepared for barcode ligation using the Ultra II End Prep reactions included in the NEBNext Companion Module for Oxford Nanopore Technologies Ligation Sequencing (Catalog # E7180S), barcoded using the NEBNext Ultra II Ligation Module (Catalog # E7595S, NEB) with ONT’s Native Barcoding Expansion 1–12 kit (EXP-NBD104). This barcoding process adds 42 min of preparation time. The ARTIC amplicons can then be pooled together for multiplexed runs, another bead clean-up is performed, and the library preparation is completed using the T4 DNA Ligase included in the NEBNext Companion Module for Oxford Nanopore Technologies Ligation Sequencing kit and the Ligation Sequencing Kit (SQK-LSK109, ONT). As an alternative, less time-consuming, and more streamlined approach to the standard ARTIC v3 protocol for preparing multiplexed libraries for nanopore sequencing, we used the Rapid Barcoding Kit (SQK-RBK004, ONT) for one-step, transposase-based fragmentation and barcoding of the two, pooled ARTIC PCR reactions for each sample (Figure 1B). The rapid adapter (RAP) is then added to the barcoded amplicons, and the final library is prepared for sequencing on the MinION. Finally, for sequencing and analysis of single samples, we pooled the two PCR reactions for each sample, performed an AMPure bead clean-up, and then used the Rapid Sequencing Kit (SQK-RAD004, ONT) for one-step, transposase-based fragmentation. The process is completed by addition of the RAP sequencing adapter and the final library is prepared for sequencing (Figure 1C).

### Sequencing

All sequencing runs were performed using either a MinION connected to a MinIT (or the Mk1C with MinKNOW 19.12 software). Each run was performed using a MinION flow cell (FLO-MIN 106 R9 version; Mk 1Spot-ON). For experiments using barcoded samples, four samples were run per flow cell. Samples that were not barcoded were run individually; one per flow cell. Prior to every run, the flow cells were assessed for the amount of total active nanopores available for sequencing, as per manufacturer’s protocol. Live basecalling with high accuracy (ONT Guppy 3.2.10) was selected for the run if the final concentration of the library was less than 10 ng/μL. If the final library concentration was too large, the basecalling would lag behind and significantly extend the time of the run. For these samples, high-accuracy basecalling was performed after the run was complete (ONT Guppy 3.2.10). For each run, the sequencing time was set to end at 20 h.

### Analysis

**FIGURE 2:**
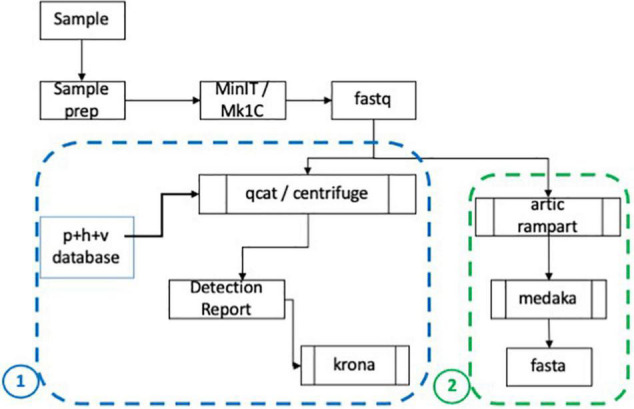
CoV-2 analysis and assembly pipeline. A schematic showing analysis of sequencing data starting with sequencing using the MinION and MinIT and creation of basecalled data (fastq) (Wang et al., 2020). The fastq files are demultiplexed if appropriate and Centrifuge is used to map reads and deliver a report of the ranked organisms and visual report (Krona). The basecalled data is also analyzed using (Wu et al., 2020) RAMPART software and the sequences with high coverage are assembled using Medaka. Fasta files are generated for subsequent analyses.

After basecalling, passed fastq files were processed by an in-house metagenomic pipeline (Figure 2, panel 1 outline). If samples were barcoded, demultiplexing was performed using qcat (ONT, version 1.0.1) with the minimum barcode quality set to 10. Centrifuge (Johns Hopkins University; Baltimore, MD, United States) was then run to align reads using Centrifuge’s pre-indexed database (h + p + v + c.tar.gz) to determine the organisms present in each sample. The Centrifuge database includes human, prokaryotic and viral genomes, and has been updated to include 106 SARS-CoV-2 genomes^2^. A report containing the top ten organisms sorted by number of reads aligned, and excluding human hits, was generated as shown in Table 2. The data was also visually compiled using Krona (National Biodefense Analysis and Countermeasures Center; Frederick, MD, United States) to produce a visual and interactive report as shown in analysis pipeline schematic (Figure 2).

**TABLE 2:** Pilot study evaluating ARTIC-based assays for sequencing SARS-CoV-2 from clinical specimens.

				Centrifuge			RAMPART					

Sample ID>	RT-PCR Diagnosis	Seq Assay	Total Reads	# CoV-2 Reads	# Unique CoV-2 Reads	% CoV-2 Reads	#CoV-2 Reads	Median Length	% CoV-2 Genome	Depth > 10x	Depth > 100x	Depth > 1,000x
BEI RNA	n/a	ARTIC v3	179,391	170,400	168,872	95.0	119,188	500	97.3	99.8	99.8	83.1
BEI RNA	n/a	ARTIC + Rapid	2,256,855	1,896,808	1,896,808	84.0	2,148,026	290	95.2	99.9	99.8	99.8
P02	+	ARTIC v3	547,474	483,403	470,900	88.3	327,577	490	77.74	98	92.9	72
P02	+	ARTIC + Rapid Barcoding	24,000	15,507	15,507	64.6	ND	ND	ND	ND	ND	ND
P02	+	ARTIC + Rapid	443,368	370,676	370,676	83.6	418,818	270	94.46	99.1	95.8	80.6
P03	+	ARTIC v3	344,910	73,885	73,867	21.4	29,429	300	15.24	56.5	20.6	15.8
P03	+	ARTIC + Rapid Barcoding	11,000	1,011	1,011	9.2	ND	ND	ND	ND	ND	ND
P03	+	ARTIC + Rapid	645,017	490,929	490,929	76.1	545,826	270.00	84.62	48.20	41.40	30.10
P04	+	ARTIC v3	543,005	119,657	118,894	22.0	39,939	310	12.2	73.1	22.6	15.8
P04	+	ARTIC + Rapid Barcoding	13,000	1,597	1,597	12.3	ND	ND	ND	ND	ND	ND
P04	+	ARTIC + Rapid	861,734	121,254	120,491	14.1	748,263	260.00	86.21	64.90	53.80	35.90
N01	-	ARTIC v3	568,266	59,246	59,246	10.4	10,482	300	3.02	82.6	22.5	4.1
N01	-	ARTIC + Rapid Barcoding	32,000	–	–	0.0	ND	ND	ND	ND	ND	ND
N01	-	ARTIC + Rapid	578,916	446,225	443,662	77.1	495,874	280	85.66	62	58.8	43.8
Neg. Ctl 1/Water	n/a	ARTIC + Rapid	25,173	7,487	7,487	29.7	8,336	190	33.11	1.5	1.4	1.3
Neg. Ctl 2/Water	n/a	ARTIC + Rapid	12,131	–	–	0.0	0	-	0	0	0	0

The ARTIC Network’s RAMPART (Read Assignment, Mapping, and Phylogenetic Analysis in Real Time) software was downloaded with instructions from: https://hub.docker.com/r/ontresearch/artic_rampart. RAMPART was used to align passed reads to the SARS-CoV-2 genome (Wuhan-Hu-1 isolate; Accession MN908947) and provided read mapping statistics and visual representations of coverage across the genome.

Additionally, bioinformatics tools for ARTIC^3^ version artic 1.1.3 were downloaded and installed inside an anaconda ver 4.8.3^4^ environment to facilitate reference based genome assembly. Assemblies with at least 89% coverage of the genome with sequencing depth of 10X were selected for whole genome assembly. Assembly was performed using a medaka^5^ based workflow for our analysis (Figure 2, panel 2 outline). The assemblies were submitted to GenBank and accession numbers are provided in Table 3. These sequences are also provided in a fasta file as Supplementary Material. Based on feedback from the GenBank submission process, we had to make corrections to P12 and P15 assemblies. There was a frameshift in P12 and upon comparison with the reference sequence at position 26,655, we found that there was an extra T after a series of 5 Ts. Since nanopore sequencing can result in errors in homopolymer regions (Watson and Warr, 2019), we corrected the assembly by removing the extra T. For P15, we found a deletion of one nucleotide in P15 sequence TTTCTTCAC was causing a frameshift and stop codons in the translation as compared to the reference sequence of TTGGCTTCAC at position 3011. This was also attributed to being error due the homopolymer region and was corrected with the addition of a T to TTTTCTTCAC.

**TABLE 3:** Assemblies with GenBank accession numbers.

ID	Seq assay	Assembly	Accession #
BEI RNA	ARTIC v3	C0V2_lsk109/ARTIC/medaka MN908947.3	ON311289
BEI RNA	ARTIC + Rapid	C0V2_rad004/ARTIC/medaka MN908947.3	ON311149
P02	ARTIC v3	1P2_lsk109/ARTIC/medaka MN908947.3	ON311288
P02	ARTIC + Rapid	1P2_rad004/ARTIC/medaka MN908947.3	ON310862
P11	ARTIC + Rapid	P11_rad004/ARTIC/medaka MN908947.3	ON310894
P12 fixed	ARTIC + Rapid	P12_rad004 (organism = Severe acute respiratory syndrome coronavirus 2)	
		(isolate = P12) reference assembly to MN908947.3	ON398848
P14	ARTIC + Rapid	P14_rad004/ARTIC/medaka MN908947.3	ON310966
P15 fixed	ARTIC + Rapid	P15_rad004 (organism = Severe acute respiratory syndrome coronavirus 2)	
		(isolate = P15) reference assembly to MN908947.3	ON398955
P18	ARTIC + Rapid	P18_rad004/ARTIC/medaka MN908947.3	ON311005

### Classification and Mutational Analyses

A FASTA file containing the six assembled genomes was uploaded to the CoVSurver app (A*STAR Bioinformatics Institute, Singapore) located on the GISAID site. The GISAID reference strain, hCOV-10/Wuhan/WIV04/2019, was used as the reference strain for comparison. The app computes and provides a list of variations and mutations in the genome. It also provides clade classification as per the GISAID classification scheme. CoV-GLUE (Singer et al., 2020), as enabled by GISAID, was used to look at the frequency of mutations in CoV-2 as observed in GISAID sequences. CoV-GLUE contains database of reported CoV-2 amino acid replacements, insertions, and deletions. Nextclade^6^ was used to generate CoV-2 clade assignments. Phylogenetic Assignment of Named Global Outbreak LINeages (pangolin, version v2.3.5, lineages version 2021-03-16^7^) was used to assign genome sequences to global CoV-2 lineages (Rambaut et al., 2020). After P12 and P15 sequences were corrected, the analyses were performed again on all the sequences using the most current versions of each of the apps: GISAID CoVsurver, Nextclade v.14.1, and Pangolin v4.0.6.

## Results

The goal of the study was to test the ARTIC CoV-2 protocol and make modifications to simplify and streamline the approach to make it faster and more accessible in its employment. We first performed a pilot, proof of concept study using a small set of previously diagnosed clinical samples. Three positive samples (P02, P03, P04) and one negative (N01), as previously diagnosed *via* EUA-cleared RT-PCR assays, were tested in this pilot study. CoV-2 RNA that was obtained from BEI Resources (NR-52285, from isolate USA-WA1/2020, Accession MN985325.1) was used as? A positive control for the assay and using the standard ARTIC protocol, 95 and 97% of 170K reads mapped to the CoV-2 genome as analyzed by Centrifuge and RAMPART, respectively (Table 2). Of note, the depth of coverage at over 100X was 99.8%, while at over 1,000X it was 83%. Over 344K total reads were obtained from each of the barcoded positive samples, but only a fraction of them mapped to CoV-2 (21–22% for P03 and P04, 88% for P02) as determined by analysis using Centrifuge and (12, 15, and 78% for P03, P04, and P02) by RAMPART. In this sample set, P02 had the most CoV-2 mapped reads (327,577), and depth of coverage of 98% at 10X as determined by RAMPART analysis. P03 and P04 had less CoV-2 mapped reads, with less of the genome covered (56.5–73.31%) at 10X depth of coverage. Unexpectedly, N01 also had several CoV-2 specific reads: 59,246 and 10,482 by Centrifuge and RAMPART, respectively. Next, we used the same amplified PCR products, but barcoded and completed sequencing library preparation using the Rapid Barcoding Kit. While this method identified CoV-2 specific reads in the positive samples by Centrifuge analysis, the number of reads returned averaged 60-fold less in comparison to the standard ARTIC v3 protocol. The ARTIC with Rapid Barcoding Kit approach did not identify any reads for the N01 sample. Finally, amplified PCR sample from the pilot samples were tested individually (and with no barcoding) *via* the ARTIC with Rapid Sequencing approach. This approach generated the most CoV-2 specific reads for all the positive samples, including the control RNA from BEI, especially as analyzed by the RAMPART pipeline. In addition, a greater percentage of the reads were mapped to CoV-2 by RAMPART (range of 84–95%) for P02-P04. The depth of coverage at over 10X for these ARTIC with Rapid sequencing positive samples were 99, 48, and 65% for P02, P03, and P04. Finally, N01 as prepared by the ARTIC with Rapid sequencing approach yielded a high number of reads (446,225 and 495,874 by Centrifuge and RAMPART, respectively) with a 62% depth of coverage at over 10X. Negative control water samples were also amplified *via* PCR and prepared using the Rapid Kit to generate background/baseline levels to expect from this assay. Two samples returned 0 and 7,487 reads by Centrifuge analysis and 0 and 8,336 by RAMPART of analysis with a depth of coverage of 1.5% at 10X depth of coverage for the negative sample that returned reads. Since the pilot study revealed that the ARTIC with Rapid sequencing approach was promising due to reduction in steps, time, and increase in CoV-2 specific reads and coverage, additional positive and negative samples were prepared and sequenced using the ARTIC with Rapid sequencing approach.

**FIGURE 3:**
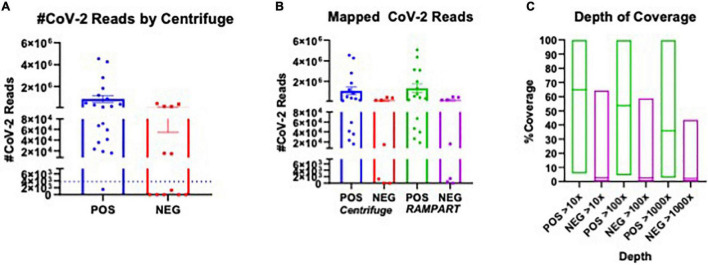
Plot of CoV-2 Reads and Coverage as analyzed using Centrifuge and RAMPART. The number of SARS-CoV2 specific reads for sequenced COVID-19 RT-PCR positive (POS) and RT-PCR negative (NEG) and genome coverage are presented. **(A)** Plot of CoV-2 reads identified using Centrifuge in all POS versus NEG samples sequenced using the ARTIC with Rapid sequencing protocol. **(B)** Comparison of POS versus NEG samples that were analyzed using both Centrifuge and RAMPART pipelines. The number of CoV-2 specific reads are shown. **(C)** The depth of coverage at > 10X, > 100X, and > 1,000X as determined by RAMPART analyses are shown for POS versus NEG samples.

**FIGURE 4:**
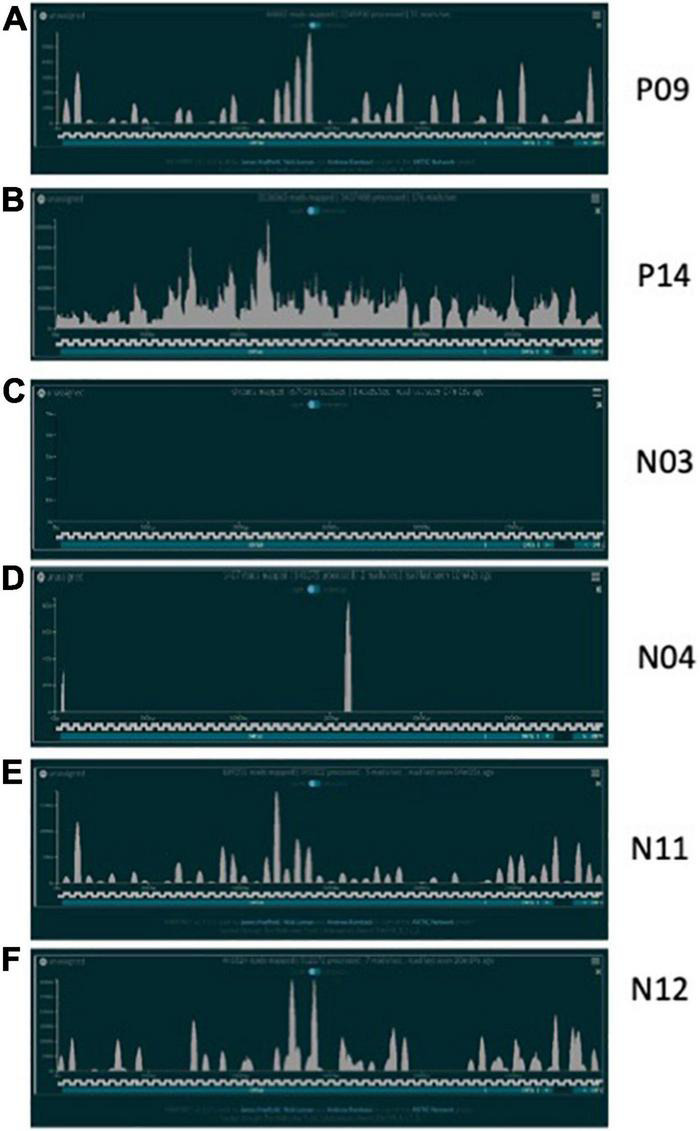
CoV-2 Reads mapped over reference genome. Representative plots showing the number of CoV-2 reads mapping over specific regions of a CoV-2 reference genome after sequencing using the ARTIC with Rapid sequencing protocol and RAMPART analyses. **(A)** P09 is shown as positive sample just below the median coverage for the positive sample cohort; **(B)** P14 is representative of high coverage samples; **(C)** N03 is a negative sample with zero CoV-2 reads identified; **(D)** N04 is a negative sample with possible non-specific amplification or contamination; **(E,F)** are two RT-PCR negative samples with 50–60% CoV-2 genome coverage following sequencing.

In total, 20 positive samples (P01-P20) and 12 negative samples (N01-N12) were sequenced using the ARTIC with Rapid sequencing approach (Table 4). The number of reads that were specifically mapped to CoV-2 as determined by Centrifuge ranged from 1,511 to 4,538,653, with a median of 150,000 in the positive sample set. The sample with the least reads was P10, which was below the estimated baseline of the assay (Table 4 and Figure 3A), but the other positive samples generated at least 15.8 K reads. For a subset of samples that were analyzed by both Centrifuge and RAMPART pipelines, CoV-2 reads ranged from 15,807 to 4,538,653 (median 336,264) using Centrifuge and 18,607–5,065,568 (median 482,322) using RAMPART. This indicated that the RAMPART pipeline was able to map more CoV-2 reads than Centrifuge (Table 4 and Figure 3B). In addition, RAMPART analysis indicated that coverage varied in these positive samples from as low as 5.7% to as high as 99.9% (median 64.9%) at > 10X depth, 4.4–99.8% (median 53.8%) at > 100X depth, and 2.7–99.8% at > 1,000X depth (Table 4 and Figure 3C). RAMPART also offers graphical representations of coverage, plotting the number of reads that map to each specific region of the CoV-2 genome (Figure 4). P09 is shown as a graphical representative of a sample with coverage just below the median (Figure 4A), while P14 represents a sample with high coverage of 99.9% (Figure 4B), both at > 10X depth of coverage.

**TABLE 4:** Sequencing of previously diagnosed clinical specimens by ARTIC with rapid sequencing approach.

				Centrifuge						RAMPART		

Sample ID	RT-PCR Diagnosis	Seq Assay	Total Reads	# CoV-2 Reads	# Unique CoV-2 Reads	% CoV-2 Reads	#CoV-2 Reads	Median Length	% CoV-2 Genome	Depth > 10x	Depth > 100x	Depth > 1,000x
P01	+	ARTIC + Rapid	462,000	71,138	71,138	15.4	ND	ND	ND	ND	ND	ND
P02	+	ARTIC + Rapid	443,368	370,676	370,676	83.6	418,818	270	94.46	99.1	95.8	80.6
P03	+	ARTIC + Rapid	645,017	490,929	490,929	76.1	545,826	270	84.62	48.2	41.4	30.1
P04	+	ARTIC + Rapid	861,734	121,254	120,491	14.1	748,263	260	86.21	64.9	53.8	35.9
P05	+	ARTIC + Rapid	44,838	18,779	18,779	41.9	ND	ND	ND	ND	ND	ND
P06	+	ARTIC + Rapid	5,136,573	4,260,587	3,808,126	82.9	4,361,013	280	84.9	99.8	99.8	98.9
P07	+	ARTIC + Rapid	1,523,922	1,189,946	1,189,171	78.1	1,331,034	290	87.34	62.2	61.3	56.2
P08	+	ARTIC + Rapid	701,282	66,782	66,782	9.5	ND	ND	ND	ND	ND	ND
P09	+	ARTIC + Rapid	2,245,950	59,114	59,114	2.6	66,862	340	2.98	57.6	42.2	18
P10	+	ARTIC + Rapid	16,691	1,511	1,511	9.1	ND	ND	ND	ND	ND	ND
P11	+	ARTIC + Rapid	3,417,719	2,816,093	2,724,325	82.4	3,087,854	290	90.35	99.8	98.3	91.5
P12	+	ARTIC + Rapid	861,623	301,852	293,972	35.0	339,009	280	60.65	89	80.2	54.5
P13	+	ARTIC + Rapid	360,276	23,244	23,244	6.5	26,335	280	7.31	7.1	6.8	4.3
P14	+	ARTIC + Rapid	3,437,488	875,159	853,443	25.5	3,136,063	290	91.23	99.9	99.8	98.2
P15	+	ARTIC + Rapid	2,100,544	1,822,242	1,754,580	86.8	1,982,939	290	94.4	97.4	96.3	82.9
P16	+	ARTIC + Rapid	237,504	15,807	15,807	6.7	18,607	280	7.83	5.7	4.4	2.7
P17	+	ARTIC + Rapid	526,781	178,745	174,792	33.9	198,615	300	37.7	25.6	24	26
P18	+	ARTIC + Rapid	5,724,659	4,538,653	4,460,075	79.3	5,065,568	280	88.49	99.9	99.8	99.8
P19	+	ARTIC + Rapid	162,340	35,574	35,574	21.9	39,440	290	24.29	38.2	34.5	7.7
P20	+	ARTIC + Rapid	286,145	41,196	41,196	14.4	46,575	230	16.28	44.1	21.4	4.6
N01	-	ARTIC + Rapid	578,916	446,225	443,662	77.1	495,874	280	85.66	62	58.8	43.8
N02	-	ARTIC + Rapid	121,813	15,313	15,313	12.6	ND	ND	ND	ND	ND	ND
N03	-	ARTIC + Rapid	67,918	–	–	0.0	–	-	0	0	0	0
N04	-	ARTIC + Rapid	141,075	1,273	1,273	0.0	1,407	290	1	2	1.9	0
N05	-	ARTIC + Rapid	649,918	–	–	0.0	431	340	0.07	1.30	1.20	0.00
N06	-	ARTIC + Rapid	168,575	–	–	0.0	ND	ND	ND	ND	ND	ND
N07	-	ARTIC + Rapid	111,549	–	–	0.0	ND	ND	ND	ND	ND	ND
N08	-	ARTIC + Rapid	30,365	–	–	0.0	ND	ND	ND	ND	ND	ND
N09	-	ARTIC + Rapid	91,432	14,671	14,656	16.0	16,219	280	17.74	2.60	2.50	2.10
N10	-	ARTIC + Rapid	574,708	183,764	183,764	32.0	208,541	280	36.29	33.90	31.70	25.50
N11	-	ARTIC + Rapid	395,322	166,603	166,603	42.1	189,251	290	47.87	64.40	56.50	33.70
N12	-	ARTIC + Rapid	512,171	386,424	386,424	75.4	441,824	270	82.26	50.30	49.70	43.30

Some unexpected results were encountered upon analysis of negative samples N01-N12. Besides the N01 sample in the pilot study, six other negative samples had reads that mapped to CoV-2 ranging from 1,273–446,225 as per analysis with Centrifuge (Table 4 and Figure 3A). Upon more careful inspection of RAMPART figures and statistics, N04 and N09 were ruled as negative because of the low number of reads (1,407 and 16,219), low depth of coverage at > 10X (2 and 2.6%), and just 2 peaks or areas of coverage on the map of the CoV-2 genome (Figure 4D for N04, Table 4). In contrast, sequencing of clinical specimens N01, N10, N11, and N12 resulted in 189,251–495,874 mapped reads, 36.29–62% depth of coverage at >10X, and multiple areas of coverage on the CoV-2 genome map (Figures 4E,F for N11 and N12, Table 4). N03 (Figure 4C) is shown in comparison as a negative clinical specimen that results in 0 CoV-2 specific reads when sequenced while clinical specimens P09 (Figure 4A) and P14 (Figure 4B) are shown as positive specimens with different percentages and areas of coverage

Next, six positive samples with relatively high genome coverage were chosen for assembly using the Wuhan-Hu-1 isolate (Accession MN908947.3) as the reference sequence. Once the sequences were successfully assembled, we used CoVsurver for clade classification and mutational analyses (Table 5). For comparison to work by others, we also included Nextstrain clade and Pango lineage classifications. As a control and reference point, we also assembled sequences generated using the CoV-2 RNA (Isolate USA-WA1/2020) from BEI that were generated using the standard Artic v3 protocol versus the Artic with Rapid modification method The comparison of the reference strain from BEI using the standard ARTIC v3 method versus the ARTIC with Rapid sequencing modification gave us the same results when performing mutational analyses using CoVsurver, as well as other lineage and classification analyses.

**TABLE 5:** Mutation analysis of assembled sequences using CoVsurver and comparison to Pangolin and Nextclade classifications.

ID	Seq Assay	Query	%N	Length (nt)	Length (aa)	#Muts	%Muts	Comment	Unique Mut	Existing Mut	GISAID Clade	Pango lineage	Nextstrain Clade
BEI RNA	ARTIC v3	C0V2_lsk109/ARTIC/medaka MN908947.3	0.00%	29,903	9,710	1	0.01%			NS8_L84S	S	A	19B
BEI RNA	ARTIC + Rapid	C0V2_rad004/ARTIC/medaka MN908947.3	0.00%	29,903	9,710	1	0.01%			NS8_L84S	S	A	19B
P02	ARTIC v3	1P2_lsk109/ARTIC/medaka MN908947.3	1.18%	29,903	9,710	4	0.04%	Stretches of NNNs (1.18% of overall sequence).		NSP12_P323L, Spike_D614G, Spike_D138Y, Spike_P521R	G	B.1.473	20A
P02	ARTIC + Rapid	1P2_rad004/ARTIC/medaka MN908947.3	2.91%	29,903	9,710	6	0.06%	Stretches of NNNs (2.91% of overall sequence).		NSP12_P323L, Spike_D614G, Spike_D138Y, Spike_P521R, NS3_Q57H, NS3_G76S	GH	B.1.473	20A
P11	ARTIC + Rapid	P11_rad004/ARTIC/medaka MN908947.3	0.00%	29,903	9,710	6	0.06%			NSP12_P323L, NSP12_H613Y, NSP14_A274S, Spike_D614G, N_G204R, N_R203K	GR	B.1.1	20B
P12	ARTIC + Rapid	P12_rad004/ARTIC/medaka MN908947.3	15.51%	29,904	9,534	6	0.06%	Long stretches of NNNs (15.51% of overall sequence). Insertion of 1 nucleotide(s) found at refpos 26653 (FRAMESHIFT). M without BLAST coverage. NSP3 has 103 Δs, NSP4 has 22 Δs; NSP16 has 80 Δs		NSP3_V477F, NSP12_P323L, Spike_D614G, Spike_P521R, NS3_Q57H, M_R44S	GH	B.1	20A
P14	ARTIC + Rapid	P14_rad004/ARTIC/medaka MN908947.3	0.00%	29,903	9,710	5	0.05%			NSP2_T85I, NSP12_P323L, Spike_D614G, NS3_Q57H, N_P364L	GH	B.1	20C
P15	ARTIC + Rapid	P15_rad004/ARTIC/medaka MN908947.3	3.30%	29,902	9,710	10	0.10%	Stretches of NNNs (3.30% of overall sequence). Gap of 1 nucleotide(s) found at refpos 3013 (FRAMESHIFT). NSP3 has 248Δs	NSP3_L98I	NSP3_A99S, NSP3_T1456I, NSP3_V477F, NSP12_P323L, Spike_D614G, Spike_V308L, Spike_P521R, NS3_Q57H, N_R209I	GH	B.1	20A
P18	ARTIC + Rapid	P18_rad004/ARTIC/medaka MN908947.3	0.00%	29,903	9,710	6	0.06%		NSP15_D212V	NSP2_T85I, NSP12_P323L, NSP13_V209I, Spike_D614G, NS3_Q57H	GH	B.1	20C
P12 fixed	ARTIC + Rapid	P12_rad004 (organism = Severe acute respiratory syndrome coronavirus 2) (isolate = P12) reference assembly to MN908947.3	15.51%	29,903	9,534	6	0.06%	Long stretches of NNNs (15.51% of overall sequence). NSP3 has 103 Δs, NSP4 has 22 Δs; NSP16 has 80 Δs		NSP3_V477F, NSP12_P323L, Spike_D614G, Spike_P521R, NS3_Q57H, M_R44S	GH	B.1	20A
P15 fixed	ARTIC + Rapid	P15_rad004 (organism = Severe acute respiratory syndrome coronavirus 2) (isolate = P15) reference assembly to MN908947.3	3.30%	29,903	9,710	10	0.10%	Stretches of NNNs (3.30% of overall sequence). NSP3 has 248Δs		NSP3_A99S, NSP3_T1456I, NSP3_V477F, NSP3_L98F, NSP12_P323L, Spike_D614G, Spike_V308L, Spike_P521R, NS3_Q57H, N_R209I	GH	B.1	20A

As expected, this isolate was assigned to GISAID clade S, and contained the NS8 L84S mutation that is one of the markers of this clade (see Table 1). We also performed this comparison for the P02 assemblies were sequencing was performed using the same standard and modified approaches. We expected similar results, but found that while the percentage of nucleotides missing (Table 5) were higher for the P02 sequenced using the ARTIC with the Rapid modification versus the standard assay, these nucleotides missing may be in 5′, 3′, or other unstranslated regions as the CoVSurver report showed only four amino acids were missing, and these were located in the NSP6 region (Supplementary Material). However, there were 26 amino acid deletions in the Spike region for the P02 sequenced using the standard ARTIC v3 assay (with LSK-109 kit). These deletions in the P02 sequenced using the standard ARTIC v3 assay may have affected the classification of this sample into clade G. Mutations in spike at D614G and P521R were also found.

For the six genomes assembled from clinical specimens that were sequenced using the ARTIC with Rapid Sequencing protocol, we identified the Spike protein D614G mutation. P02, P12, P14, P15, and P18 were classified in clade GH. P11 was classified into clade GR, which diverged after GH, and deviates from GH in that it carries the N R203K mutation, but not the NS3 Q57H mutation (see Tables 1, 5). Overall, the number of mutations as compared to the WIV04 reference isolate were minimal ranging from 3 to 7 amino acid changes (0.05–0.10%). There were a number of mutations that had been previously observed by others, more notably, the Spike D614G, Nsp12 P323L, and NS3 Q57H mutations. There were also five mutations that were unique to these samples when we first analyzed them using CoVsurver on October 20, 2020: Spike P521R, NS3 G76S, N P364L, Nsp13 V209I, and Nsp15 D212V. Of note, the Spike P521R mutation was observed in 3 of these assemblies.

## Discussion

It is not surprising that an RNA virus would mutate over time, especially during an extended period of human-to-human transmission as has been the case with the pandemic caused by SARS-CoV-2. One important mutation that arose early on was the D614G mutation in the Spike protein, and this mutation quickly becoming prevalent upon its introduction or emergence in different areas of the world (Korber et al., 2020). Of note, while the rate of mutation in the CoV-2, including in the Spike protein was low at the time, the D614G mutation was caused by a single nucleotide mutation from A-to-G at position 23,403 in the Wuhan reference strain (Korber et al., 2020). Our sequence assemblies all carried this substitution D614G in the Spike protein, and the P323L mutation in the Nsp12 (RNA-dependent RNA polymerase) that frequently co-evolves with it (Coppee et al., 2020). GISAID and others began to track the mutation in March 2020, and the clade carrying the substitution was designated as G. Within that month, the variant went from being present in 10% of global sequences, to 67% of global sequences (Korber et al., 2020). Given the speed at which the D614G variant was able to spread, higher Ct counts in patients (Korber et al., 2020), and higher infectivity of VSV-pseudotyped virions (Korber et al., 2020; Li et al., 2020), the mutation may offer a fitness advantage that makes the virus more infectious. Furthermore, other important variants with increased transmissibility continued to emerge that carried the D614G mutation, along with a number of other mutations. These variants emerged in the fall of 2020 and were reported by the United Kingdom (B.1.1.7, WHO designation Alpha, GISAID Clade GRY), South Africa (B.1.351), and Brazil (P.1, WHO designation Gamma) (CDC, 2021a). We also found a P521R mutation in the Spike RBD in three of our six assemblies. The GISAID site reports that this mutation was first reported in March 2020 as found in an Israeli strain: hCoV-19/Israel,CVL-n2487/2020. Additional analyses on sequences containing this mutation as reported by CoV-GLUE confirm the mutation’s presence in New Mexico and Arizona from strains collected in April 2020 to June 2020. Skipping a year forward, and there was the emergence and eventual global dominance of the WHO label Delta variant (B.1.617.2, GISAID clade GK), that carried 15 substitutions or deletions in the Spike protein including the aforementioned D614G (CDC, 2021b). Once again, this newer variant was observed to have increased transmissibility, with reduction in neutralization by post-vaccination sera was observed. By the November 2021, South Africa had reported and identified yet a new variant that contained at least 30 amino acid substitutions, 3 deletions and an insertion in the Spike protein alone. This new variant was designated as the Omicron variant of concern by the WHO (B.1.1.529, GISAID clade GRA) and while vaccines are still considered to be effective in preventing severe illness and hospitalizations, break-through infections are observed (CDC, 2021c).

Besides the D614G and P323L mutations that as previously mentioned have a tendency to co-evolve, another frequent mutation observed in our assembled genomes was ORF3a or NS3 Q57H (P02, P14, P15, P18, and P12 once corrected). Coppée et al. reported this mutation as the fourth frequent mutation observed in European populations after D164G, P323L, and L84S as accessed in sequences collected through April 17, 2020. The Q57H mutation was frequent in samples from France and Belgium, but not Italy and Spain (Coppee et al., 2020). By November 2020, the introduction of a GISAID clade GH virus with this G57H mutation had lead to a fourth wave of CoV-2 infections in Hong Kong (Chu et al., 2021). While they did not find enhanced replication kinetics or increased induction of cytokines/chemokines by this virus (Chu et al., 2021), a different study showed that the Q57H mutation increased the intraviral protein affinities (Wu et al., 2021). Moreover, while Q57 was not involved in protein-binding interfaces, Q57H was a hotspot for protein-interactions (Wu et al., 2021). The emergence of Q57H earlier on during the pandemic as reported here and by others, and more recent reemergence associated with a wave of infections may suggest an advantage to the virus. Besides the Q57H mutation, we also found a mutation in NS3 that was unique to our subset: G76S.

Other unique mutations to our assemblies included those in the multidomain, multifunctional Nsp3 protein (Santerre et al., 2020) in sample P15: A99S and L98I or L98F. However, because the Nsp3 region in this assembly had significant gaps, it is difficult to ascertain that these mutations are real. In one of our better assemblies, using sample P18, we found mutations in the helicase Nsp13 V209I and endoribonuclease Nsp15 NSP15D212V.

A subset of negative samples, as determined by PCR analyses returned hits that mapped to SARS-CoV-2 as analyzed by Centrifuge and RAMPART. A caveat of this study is that we did not have detailed PCR assay information with the samples, including which PCR test was used or Ct values. However, the PCR tests with EUA at the time from April to May 2020 consisted of single to three target site assays (FDA, 2022). Since the ARTIC primers span the entire genome, the assay is likely more sensitive, and is likely to pick up samples that were found to be negative by the assays. For clinical specimens N01, N10, N11, and N12 we had 189,251–495,874 mapped reads, and multiple areas of coverage on the CoV-2 genome map (Figure 4), and thus it is possible that these specimens were in fact positive, but missed by the one to three-target PCR tests. At the same time, because of the high number of primers/targets and amplification cycles that we used, there was also a chance for non-specific amplification as may have been the case with N04 and N09, which have low read numbers, and very little coverage of the CoV-2 genome. Thus, we don’t think that N04 and N09 are positive for CoV-2. On the other hand, positive sample P10 seemed to generate very few reads, and even fewer CoV-2 specific reads similar to subject N04. Because we don’t have corresponding PCR information, including Ct value, we can’t rule out if this was a false positive, a sample with a very low viral load, or a sample that got degraded after processing and shipment to our lab.

Non-specific or contaminating reads that we observed in the samples as reported by Centrifuge mapped to *Escheria coli*, *Shigella boydii*, *Shigella flexneri*, *Shigella phage*, and *Escheria phage Mu* as examples. These reads may be related to contaminating bacterial DNA derived from the extraction kit or library preparation kits. These hits were associated with a low percentage of unique reads (e.g., *E. coli* 6.5%, Escheria phage Mu 0.1%). There were also reads that failed to map using Centrifuge, and these could have been reads associated with the host (human). However, human/host reads can now be filtered out during sequencing using “adaptive sequencing” from MinKNOW and selecting for depletion of human sequences. This is an approach that may be used in future studies.

In short, the work presented shows that it is possible to streamline the Artic v3 protocol for nanopore sequencing, saving an hour and 10 min in sample and library preparation time (Figure 1),and still acquire data for sensitive and confident identification of CoV-2 in human nasopharyngeal swab samples. For a subset of samples, presumably for those with ample viral loads, whole genome assembly was possible. Future studies will investigate what additional methods can be used to improve results, for example, use of primers that generate longer reads might improve results. One caveat of the current approach is that the Rapid Sequencing Kit was used to cleave the 400 b amplicons generated using the ARTIC v3 primer panel. This could result in failed sequences since the shorter reads may be misidentified as adapters during acquisition or may be flagged as failed reads by the basecaller. However, from the samples that were assembled, we were able to find and confirm deviations that were previously reported by others, and that were consistent with geographic location and period in which these samples were collected. Moreover, we were able to identify a few mutations that were unique to these samples. This work suggests that accessible, portable, and relatively fast sample-to-sequence processes can be effectively used to characterize viral outbreaks. Processes like this are needed to bring sequencing and characterization to the initial sites of emerging outbreaks, even if they are occurring in remote regions, and will help us be better prepared to respond. There are additional modifications that could make this approach more field-friendly. One modification would be to move from RNA extraction kits that use Trizol and require centrifugation with benchtop instruments to kits that use a different lysis buffer and can use either a magnetic, RNA-binding bead-based method or a syringe-based silica-based column method. Adding an automated software package that can perform analysis in real time as the sample is being sequenced would also help expedite the process by providing faster identification of the sample. If the sample of interest is positive, additional sequencing time can be used to generate enough reads for further characterization and assembly. Another time-saving modification could be focusing on a single area of interest such as the spike protein gene to simplify the library preparation process and reduce analysis time. Although this would further streamline the approach, it would not be as powerful as whole genome sequencing that would provide further insight as to how the whole virus is evolving. Furthermore, as this virus continues to mutate and affect the global population, an all-hands/all-methods approach to surveillance may be needed to finally get ahead of the curve.

## Data Availability Statement

The data presented in the study are deposited in the NCBI GenBank repository (https://www.ncbi.nlm.nih.gov/nuccore/), with accession numbers provided in Table 3.

## Author Contributions

MA and RB conceived the study, design, and experiments. MA, MK, SK, and JVH performed experiments. JMH, SD, PR, and AL performed bioinformatic analysis and assembly of the sequences. MA performed classification and mutational analyses. MA prepared the manuscript with input, review, and contributions provided by MK, SK, JVH, JMH, SD, PR, AL, and RB. All authors contributed to the article and approved the submitted version.

## Conflict of Interest

JMH, PR, and AL were employed by DCS Corporation. The remaining authors declare that the research was conducted in the absence of any commercial or financial relationships that could be construed as a potential conflict of interest.

## Publisher’s Note

All claims expressed in this article are solely those of the authors and do not necessarily represent those of their affiliated organizations, or those of the publisher, the editors and the reviewers. Any product that may be evaluated in this article, or claim that may be made by its manufacturer, is not guaranteed or endorsed by the publisher.

## Footnotes

1
https://www.gisaid.org/

2
https://ccb.jhu.edu/software/centrifuge/index.shtml

3
https://github.com/artic-network/fieldbioinformatics

4
www.anaconda.com

5
https://github.com/nanoporetech/medaka

6
clades.nextstrain.org

7
https://pangolin.cog-uk.io/
