# Supplementary material for: A Rapid, Whole Genome Sequencing Assay for Detection and Characterization of Novel Coronavirus (SARS-CoV-2) Clinical Specimens Using Nanopore Sequencing
Source: Front Microbiol. 2022 Jun 6;13:910955. doi: 10.3389/fmicb.2022.910955 (PMC9207459; doi:10.3389/fmicb.2022.910955)
Supplement: Supplementary file 2 [file Data_Sheet_2.PDF]

The main application scenario for CoVsurver is to highlight phenotypically or epidemiologically interesting candidate amino acid (aa) changes for further research and should ideally be combined with experimental testing and verification of any predicted phenotypes.

Result for comparison with reference selection: **hCoV-19/Wuhan/WIV04/2019**

3D structural visualization of the spike glycoprotein with aa changes identified in the query sequences shown as colored balls

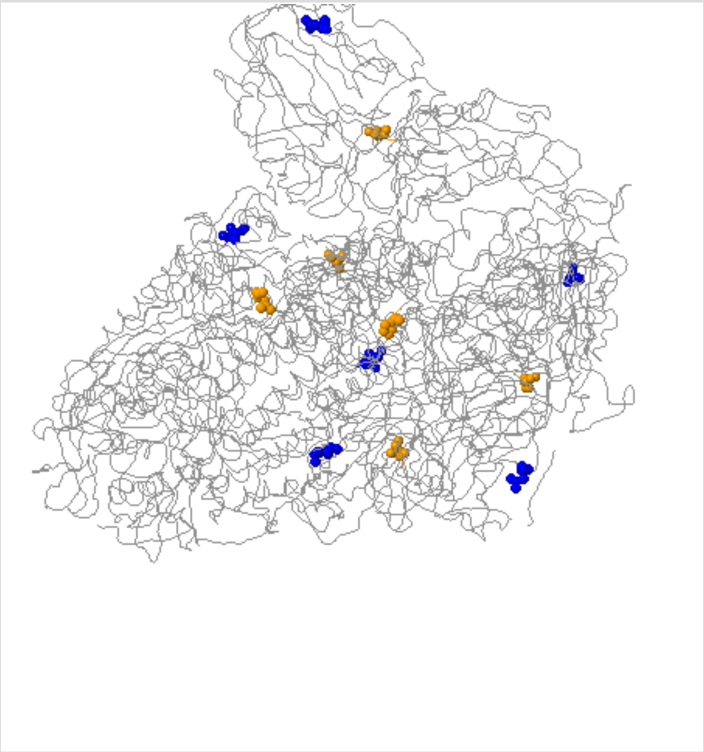

Spin ON Spin OFF Save IMAGE

Spike glycoprotein (PDB: 6acc, EM 3.6 Angstrom) with RBD in down conformation.

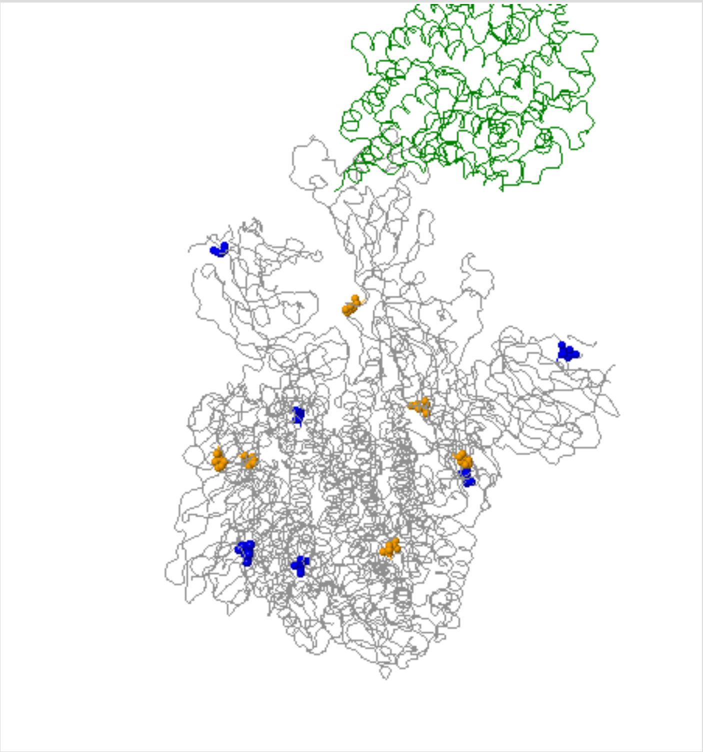

Spin ON Spin OFF Save IMAGE

Spike glycoprotein (PDB: 6acj, EM 4.2 Angstrom) in complex with host cell receptor ACE2 (green ribbon).

% AA identity: 99.686%

# aa changes: 4

List of variations displayed in structure (nearest residue if in loop/termini region)

D138Y V308L P521R D614G

Query	Clade	Best reference hit	%id	%coverag	#Δs	List of aa changes
		NSP1 hCoV-19/Wuhan/WIV04/2019	100%	100%	0	no aa changes
		NSP2 hCoV-19/Wuhan/WIV04/2019	100%	100%	0	no aa changes
		NSP3 hCoV-19/Wuhan/WIV04/2019	100%	49.7%	0	no aa changes
		NSP4 hCoV-19/Wuhan/WIV04/2019	100%	100%	0	no aa changes
		NSP5 hCoV-19/Wuhan/WIV04/2019	100%	100%	0	no aa changes
		NSP6 hCoV-19/Wuhan/WIV04/2019	100%	100%	0	no aa changes
		NSP7 hCoV-19/Wuhan/WIV04/2019	100%	100%	0	no aa changes
		NSP8 hCoV-19/Wuhan/WIV04/2019	100%	100%	0	no aa changes
		NSP9 hCoV-19/Wuhan/WIV04/2019	100%	100%	0	no aa changes
		NSP10 hCoV-19/Wuhan/WIV04/2019	100%	100%	0	no aa changes
		NSP11 hCoV-19/Wuhan/WIV04/2019	100%	100%	0	no aa changes
		NSP12 hCoV-19/Wuhan/WIV04/2019	99.9%	99.0%	1	**P323L**[#o]
		NSP13 hCoV-19/Wuhan/WIV04/2019	100%	100%	0	no aa changes
		NSP14 hCoV-19/Wuhan/WIV04/2019	100%	100%	0	no aa changes
1P2_lsk109/ARTIC/medaka MN908947.3	G	NSP15 hCoV-19/Wuhan/WIV04/2019	100%	100%	0	no aa changes
		NSP16 hCoV-19/Wuhan/WIV04/2019	100%	100%	0	no aa changes
						**D138Y**, **S151X**[$#a], **W152X**[#a], **M153X**, **E154X**, **S155X**,

		Spike hCoV-19/Wuhan/WIV04/2019	97.8%	100%	28	**E156X**, **F157X**, **R158X**[#a], **V227X**[#o], **D228X**[#lo], **L229X**[#lo], **P230X**[#lo], **I231X**[#o], **G232X**[#lo], **I233X**[#lo], **N234X**[$#lo], **I235X**[#lo], **T236X**[#o], **R237X**, **F238X**, **Q239X**[$], **T240X**, **L241X**, **L242X**[$], **A243X**[$], **P521R**[$#o], **D614G**[$#lo]
		NS3 hCoV-19/Wuhan/WIV04/2019	99.3%	100%	2	**Q57X**[#o], **G76X**[$]
		E hCoV-19/Wuhan/WIV04/2019	100%	100%	0	no aa changes
		M hCoV-19/Wuhan/WIV04/2019	100%	100%	0	no aa changes
		NS6 hCoV-19/Wuhan/WIV04/2019	100%	100%	0	no aa changes
		NS7a hCoV-19/Wuhan/WIV04/2019	100%	100%	0	no aa changes
		NS7b hCoV-19/Wuhan/WIV04/2019	100%	100%	0	no aa changes
		NS8 hCoV-19/Wuhan/WIV04/2019	100%	100%	0	no aa changes
		N hCoV-19/Wuhan/WIV04/2019	100%	100%	0	no aa changes
		NSP1 hCoV-19/Wuhan/WIV04/2019	100%	100%	0	no aa changes
		NSP2 hCoV-19/Wuhan/WIV04/2019	100%	100%	0	no aa changes
		NSP3 hCoV-19/Wuhan/WIV04/2019	100%	49.7%	0	no aa changes
		NSP4 hCoV-19/Wuhan/WIV04/2019	100%	76.0%	0	no aa changes
		NSP5 hCoV-19/Wuhan/WIV04/2019	100%	100%	0	no aa changes
		NSP6 hCoV-19/Wuhan/WIV04/2019	98.6%	100%	4	**L14X**, **L15X**, **L16X**, **T17X**
		NSP7 hCoV-19/Wuhan/WIV04/2019	100%	100%	0	no aa changes
		NSP8 hCoV-19/Wuhan/WIV04/2019	100%	100%	0	no aa changes
		NSP9 hCoV-19/Wuhan/WIV04/2019	100%	100%	0	no aa changes
		NSP10 hCoV-19/Wuhan/WIV04/2019	100%	100%	0	no aa changes
		NSP11 hCoV-19/Wuhan/WIV04/2019	100%	100%	0	no aa changes
		NSP12 hCoV-19/Wuhan/WIV04/2019	99.9%	99.0%	1	**P323L**[#o]
1P2_rad004/ARTIC/medaka MN908947.3	GH	NSP13 hCoV-19/Wuhan/WIV04/2019	100%	100%	0	no aa changes
		NSP14 hCoV-19/Wuhan/WIV04/2019	100%	100%	0	no aa changes
		NSP15 hCoV-19/Wuhan/WIV04/2019	100%	100%	0	no aa changes
		NSP16 hCoV-19/Wuhan/WIV04/2019	100%	100%	0	no aa changes
		Spike hCoV-19/Wuhan/WIV04/2019	99.8%	80.1%	2	**P521R**[$#o], **D614G**[$#lo]
		NS3 hCoV-19/Wuhan/WIV04/2019	99.3%	100%	2	**Q57H**[#o], **G76S**[$]
		E hCoV-19/Wuhan/WIV04/2019	100%	100%	0	no aa changes
		M hCoV-19/Wuhan/WIV04/2019	100%	100%	0	no aa changes
		NS6 hCoV-19/Wuhan/WIV04/2019	100%	100%	0	no aa changes
		NS7a hCoV-19/Wuhan/WIV04/2019	100%	100%	0	no aa changes
		NS7b hCoV-19/Wuhan/WIV04/2019	100%	100%	0	no aa changes
		NS8 hCoV-19/Wuhan/WIV04/2019	100%	100%	0	no aa changes
		N hCoV-19/Wuhan/WIV04/2019	100%	100%	0	no aa changes
		NSP1 hCoV-19/Wuhan/WIV04/2019	100%	100%	0	no aa changes
		NSP2 hCoV-19/Wuhan/WIV04/2019	100%	100%	0	no aa changes
		NSP3 hCoV-19/Wuhan/WIV04/2019	100%	100%	0	no aa changes
		NSP4 hCoV-19/Wuhan/WIV04/2019	100%	100%	0	no aa changes
		NSP5 hCoV-19/Wuhan/WIV04/2019	100%	100%	0	no aa changes
		NSP6 hCoV-19/Wuhan/WIV04/2019	100%	100%	0	no aa changes
		NSP7 hCoV-19/Wuhan/WIV04/2019	100%	100%	0	no aa changes
		NSP8 hCoV-19/Wuhan/WIV04/2019	100%	100%	0	no aa changes
		NSP9 hCoV-19/Wuhan/WIV04/2019	100%	100%	0	no aa changes
		NSP10 hCoV-19/Wuhan/WIV04/2019	100%	100%	0	no aa changes
		NSP11 hCoV-19/Wuhan/WIV04/2019	100%	100%	0	no aa changes
		NSP12 hCoV-19/Wuhan/WIV04/2019	100%	99.0%	0	no aa changes
C0V2_lsk109/ARTIC/medaka MN908947.3	S	NSP13 hCoV-19/Wuhan/WIV04/2019	100%	100%	0	no aa changes
		NSP14 hCoV-19/Wuhan/WIV04/2019	100%	100%	0	no aa changes
		NSP15 hCoV-19/Wuhan/WIV04/2019	100%	100%	0	no aa changes
		NSP16 hCoV-19/Wuhan/WIV04/2019	100%	100%	0	no aa changes
		Spike hCoV-19/Wuhan/WIV04/2019	100%	100%	0	no aa changes
		NS3 hCoV-19/Wuhan/WIV04/2019	100%	100%	0	no aa changes
		E hCoV-19/Wuhan/WIV04/2019	100%	100%	0	no aa changes
		M hCoV-19/Wuhan/WIV04/2019	100%	100%	0	no aa changes

		NS6 hCoV-19/Wuhan/WIV04/2019	100%	100%	0	no aa changes
		NS7a hCoV-19/Wuhan/WIV04/2019	100%	100%	0	no aa changes
		NS7b hCoV-19/Wuhan/WIV04/2019	100%	100%	0	no aa changes
		NS8 hCoV-19/Wuhan/WIV04/2019	99.2%	100%	1	L84S
		N hCoV-19/Wuhan/WIV04/2019	100%	100%	0	no aa changes
		NSP1 hCoV-19/Wuhan/WIV04/2019	100%	100%	0	no aa changes
		NSP2 hCoV-19/Wuhan/WIV04/2019	100%	100%	0	no aa changes
		NSP3 hCoV-19/Wuhan/WIV04/2019	100%	100%	0	no aa changes
		NSP4 hCoV-19/Wuhan/WIV04/2019	100%	100%	0	no aa changes
		NSP5 hCoV-19/Wuhan/WIV04/2019	100%	100%	0	no aa changes
		NSP6 hCoV-19/Wuhan/WIV04/2019	100%	100%	0	no aa changes
		NSP7 hCoV-19/Wuhan/WIV04/2019	100%	100%	0	no aa changes
		NSP8 hCoV-19/Wuhan/WIV04/2019	100%	100%	0	no aa changes
		NSP9 hCoV-19/Wuhan/WIV04/2019	100%	100%	0	no aa changes
		NSP10 hCoV-19/Wuhan/WIV04/2019	100%	100%	0	no aa changes
		NSP11 hCoV-19/Wuhan/WIV04/2019	100%	100%	0	no aa changes
		NSP12 hCoV-19/Wuhan/WIV04/2019	100%	99.0%	0	no aa changes
C0V2_rad004/ARTIC/medaka MN908947.3	S	NSP13 hCoV-19/Wuhan/WIV04/2019	100%	100%	0	no aa changes
		NSP14 hCoV-19/Wuhan/WIV04/2019	100%	100%	0	no aa changes
		NSP15 hCoV-19/Wuhan/WIV04/2019	100%	100%	0	no aa changes
		NSP16 hCoV-19/Wuhan/WIV04/2019	100%	100%	0	no aa changes
		Spike hCoV-19/Wuhan/WIV04/2019	100%	100%	0	no aa changes
		NS3 hCoV-19/Wuhan/WIV04/2019	100%	100%	0	no aa changes
		E hCoV-19/Wuhan/WIV04/2019	100%	100%	0	no aa changes
		M hCoV-19/Wuhan/WIV04/2019	100%	100%	0	no aa changes
		NS6 hCoV-19/Wuhan/WIV04/2019	100%	100%	0	no aa changes
		NS7a hCoV-19/Wuhan/WIV04/2019	100%	100%	0	no aa changes
		NS7b hCoV-19/Wuhan/WIV04/2019	100%	100%	0	no aa changes
		NS8 hCoV-19/Wuhan/WIV04/2019	99.2%	100%	1	L84S
		N hCoV-19/Wuhan/WIV04/2019	100%	100%	0	no aa changes
		NSP1 hCoV-19/Wuhan/WIV04/2019	100%	100%	0	no aa changes
		NSP2 hCoV-19/Wuhan/WIV04/2019	100%	100%	0	no aa changes
		NSP3 hCoV-19/Wuhan/WIV04/2019	100%	100%	0	no aa changes
		NSP4 hCoV-19/Wuhan/WIV04/2019	100%	100%	0	no aa changes
		NSP5 hCoV-19/Wuhan/WIV04/2019	100%	100%	0	no aa changes
		NSP6 hCoV-19/Wuhan/WIV04/2019	100%	100%	0	no aa changes
		NSP7 hCoV-19/Wuhan/WIV04/2019	100%	100%	0	no aa changes
		NSP8 hCoV-19/Wuhan/WIV04/2019	100%	100%	0	no aa changes
		NSP9 hCoV-19/Wuhan/WIV04/2019	100%	100%	0	no aa changes
		NSP10 hCoV-19/Wuhan/WIV04/2019	100%	100%	0	no aa changes
		NSP11 hCoV-19/Wuhan/WIV04/2019	100%	100%	0	no aa changes
		NSP12 hCoV-19/Wuhan/WIV04/2019	99.8%	99.0%	2	P323L[#o], H613Y
P11_rad004/ARTIC/medaka MN908947.3	GR	NSP13 hCoV-19/Wuhan/WIV04/2019	100%	100%	0	no aa changes
		NSP14 hCoV-19/Wuhan/WIV04/2019	99.8%	100%	1	A274S
		NSP15 hCoV-19/Wuhan/WIV04/2019	100%	100%	0	no aa changes
		NSP16 hCoV-19/Wuhan/WIV04/2019	100%	100%	0	no aa changes
		Spike hCoV-19/Wuhan/WIV04/2019	99.9%	100%	1	D614G[$#lo]
		NS3 hCoV-19/Wuhan/WIV04/2019	100%	100%	0	no aa changes
		E hCoV-19/Wuhan/WIV04/2019	100%	100%	0	no aa changes
		M hCoV-19/Wuhan/WIV04/2019	100%	100%	0	no aa changes
		NS6 hCoV-19/Wuhan/WIV04/2019	100%	100%	0	no aa changes
		NS7a hCoV-19/Wuhan/WIV04/2019	100%	100%	0	no aa changes
		NS7b hCoV-19/Wuhan/WIV04/2019	100%	100%	0	no aa changes
		NS8 hCoV-19/Wuhan/WIV04/2019	100%	100%	0	no aa changes
		N hCoV-19/Wuhan/WIV04/2019	99.5%	100%	2	R203K, G204R
		NSP1 hCoV-19/Wuhan/WIV04/2019	100%	100%	0	no aa changes

P12_rad004/ARTIC/medaka MN908947.3	GH	NSP2 hCoV-19/Wuhan/WIV04/2019	100%	100%	0	no aa changes
		NSP3 hCoV-19/Wuhan/WIV04/2019	89.3%	49.7%	103	S370X[#o], S371X[#o], F372X[#o], L373X[#o], E374X[#o], M375X, K376X, S377X, E378X, K379X, Q380X, V381X, E382X, Q383X, K384X, I385X, A386X, E387X, I388X, P389X, K390X, E391X, E392X, V393X, K394X, P395X, F396X, I397X, T398X, E399X, S400X, K401X, P402X, S403X, V404X, E405X, Q406X, R407X, K408X, Q409X, D410X, D411X, K412X, K413X, I414X, K415X[#o], A416X, C417X, V418X, E419X, E420X[#o], V421X[#o], T422X, T423X[#o], T424X, L425X, E426X, E427X, T428X[#o], K429X[#o], F430X, L431X[#o], T432X[#o], E433X[#o], N434X[#o], L435X, L436X, L437X, Y438X, I439X, D440X, I441X[#o], N442X[#o], G443X, N444X, L445X, H446X, P447X, D448X, S449X, A450X, T451X, L452X, V453X, V477F[#o], I759X[#ho], N760X[#ho], L761X[#ho], H762X[#ho], T763X[#ho], Q764X[#o], V765X[#ho], V766X, D767X[#o], M768X, S769X[#o], M770X[#o], T771X[#o], Y772X[#o], G773X[#o], Q774X[#o], Q775X[#o], F776X[#o]
		NSP4 hCoV-19/Wuhan/WIV04/2019	95.6%	100%	22	T28X, P29X, V30X, H31X, V32X, M33X, S34X, K35X, H36X, T37X, D38X, F39X, R112X, T113X, T114X, N115X, G116X, D117X, F118X, L119X, H120X, T215X
		NSP5 hCoV-19/Wuhan/WIV04/2019	99.7%	100%	1	G120X[#o]
		NSP6 hCoV-19/Wuhan/WIV04/2019	99.7%	100%	1	L69X
		NSP7 hCoV-19/Wuhan/WIV04/2019	100%	33.7%	0	no aa changes
		NSP8 hCoV-19/Wuhan/WIV04/2019	100%	92.9%	0	no aa changes
		NSP9 hCoV-19/Wuhan/WIV04/2019	100%	100%	0	no aa changes
		NSP10 hCoV-19/Wuhan/WIV04/2019	100%	100%	0	no aa changes
		NSP11 hCoV-19/Wuhan/WIV04/2019	100%	100%	0	no aa changes
		NSP12 hCoV-19/Wuhan/WIV04/2019	99.9%	74.0%	1	P323L[#o]
		NSP13 hCoV-19/Wuhan/WIV04/2019	100%	99.8%	0	no aa changes
		NSP14 hCoV-19/Wuhan/WIV04/2019	99.2%	23.5%	1	G123X
		NSP15 hCoV-19/Wuhan/WIV04/2019	100%	100%	0	no aa changes
		NSP16 hCoV-19/Wuhan/WIV04/2019	73.2%	100%	80	G164X, G165X, S166X, V167X, A168X, I169X, K170X, I171X, T172X, E173X, H174X, S175X, W176X, N177X, A178X, D179X, L180X, Y181X, K182X, L183X, M184X, G185X, H186X, F187X, A188X, W189X, W190X, T191X, A192X, F193X, V194X, T195X, N196X, V197X, N198X, A199X, S200X, S201X, S202X, E203X, A204X, F205X, L206X, I207X, G208X, C209X, N210X, Y211X, L212X, G213X, K214X, P215X, R216X, E217X, Q218X, I219X, D220X, G221X, Y222X, V223X, M224X, H225X, A226X, N227X, Y228X, I229X, F230X, W231X, R232X, N233X, T234X, N235X, P236X, I237X, Q238X, L239X, S240X, S241X, Y242X, S243X
		Spike hCoV-19/Wuhan/WIV04/2019	99.5%	33.5%	2	P521R[$#o], D614G[$#lo]
		NS3 hCoV-19/Wuhan/WIV04/2019	100%	35.3%	0	no aa changes
		E hCoV-19/Wuhan/WIV04/2019	100%	100%	0	no aa changes
		(no ref hits for M)	0%	0%	0	no coverage
		NS6 hCoV-19/Wuhan/WIV04/2019	100%	100%	0	no aa changes
		NS7a hCoV-19/Wuhan/WIV04/2019	100%	92.6%	0	no aa changes
		NS7b hCoV-19/Wuhan/WIV04/2019	100%	58.1%	0	no aa changes
		NS8 hCoV-19/Wuhan/WIV04/2019	100%	100%	0	no aa changes
		N hCoV-19/Wuhan/WIV04/2019	100%	66.1%	0	no aa changes
		NSP1 hCoV-19/Wuhan/WIV04/2019	100%	100%	0	no aa changes
		NSP2 hCoV-19/Wuhan/WIV04/2019	99.8%	100%	1	T85I
		NSP3 hCoV-19/Wuhan/WIV04/2019	100%	100%	0	no aa changes
		NSP4 hCoV-19/Wuhan/WIV04/2019	100%	100%	0	no aa changes
		NSP5 hCoV-19/Wuhan/WIV04/2019	100%	100%	0	no aa changes
		NSP6 hCoV-19/Wuhan/WIV04/2019	100%	100%	0	no aa changes
		NSP7 hCoV-19/Wuhan/WIV04/2019	100%	100%	0	no aa changes
		NSP8 hCoV-19/Wuhan/WIV04/2019	100%	100%	0	no aa changes
		NSP9 hCoV-19/Wuhan/WIV04/2019	100%	100%	0	no aa changes
		NSP10 hCoV-19/Wuhan/WIV04/2019	100%	100%	0	no aa changes
		NSP11 hCoV-19/Wuhan/WIV04/2019	100%	100%	0	no aa changes

P14_rad004/ARTIC/medaka MN908947.3	GH	NSP12 hCoV-19/Wuhan/WIV04/2019	99.9%	99.0%	1	P323L[#o]
		NSP13 hCoV-19/Wuhan/WIV04/2019	100%	100%	0	no aa changes
		NSP14 hCoV-19/Wuhan/WIV04/2019	100%	100%	0	no aa changes
		NSP15 hCoV-19/Wuhan/WIV04/2019	100%	100%	0	no aa changes
		NSP16 hCoV-19/Wuhan/WIV04/2019	100%	100%	0	no aa changes
		Spike hCoV-19/Wuhan/WIV04/2019	99.9%	100%	1	D614G[$#lo]
		NS3 hCoV-19/Wuhan/WIV04/2019	99.6%	100%	1	Q57H[#o]
		E hCoV-19/Wuhan/WIV04/2019	100%	100%	0	no aa changes
		M hCoV-19/Wuhan/WIV04/2019	100%	100%	0	no aa changes
		NS6 hCoV-19/Wuhan/WIV04/2019	100%	100%	0	no aa changes
		NS7a hCoV-19/Wuhan/WIV04/2019	100%	100%	0	no aa changes
		NS7b hCoV-19/Wuhan/WIV04/2019	100%	100%	0	no aa changes
		NS8 hCoV-19/Wuhan/WIV04/2019	100%	100%	0	no aa changes
		N hCoV-19/Wuhan/WIV04/2019	99.8%	100%	1	P364L[$#o]
P15_rad004/ARTIC/medaka MN908947.3	GH	NSP1 hCoV-19/Wuhan/WIV04/2019	100%	100%	0	no aa changes
		NSP2 hCoV-19/Wuhan/WIV04/2019	99.8%	76.0%	1	S299X
		NSP3 hCoV-19/Wuhan/WIV04/2019	86.6%	95.0%	248	L98I, A99S, S370X[#o], S371X[#o], F372X[#o], L373X[#o], E374X[#o], M375X, K376X, S377X, E378X, K379X, Q380X, V381X, E382X, Q383X, K384X, I385X, A386X, E387X, I388X, P389X, K390X, E391X, E392X, V393X, K394X, P395X, F396X, I397X, T398X, E399X, S400X, K401X, P402X, S403X, V404X, E405X, Q406X, R407X, K408X, Q409X, D410X, D411X, K412X, K413X, I414X, K415X[#o], A416X, C417X, V418X, E419X, E420X[#o], V421X[#o], T422X, T423X[#o], T424X, L425X, E426X, E427X, T428X[#o], K429X[#o], F430X, L431X[#o], T432X[#o], E433X[#o], N434X[#o], L435X, L436X, L437X, Y438X, I439X, D440X, I441X[#o], N442X[#o], G443X, N444X, L445X, H446X, P447X, D448X, S449X, A450X, T451X, L452X, V453X, V477F[#o], Y1055X[#o], T1056X[#o], T1057X[#o], T1058X[#o], I1059X[#o], N1177X, N1178X, A1179X, T1180X, N1181X, K1182X, A1183X, T1184X, Y1185X, K1186X, P1187X, N1188X, T1189X, W1190X, C1191X, I1192X, R1193X, C1194X, L1195X, W1196X, S1197X, T1198X, K1199X, P1200X, V1201X, E1202X, T1203X, S1204X, N1205X, S1206X, F1207X, D1208X, V1209X, L1210X, K1211X, S1212X, E1213X, D1214X, A1215X, Q1216X, G1217X, M1218X, D1219X, N1220X, L1221X, A1222X, C1223X, E1224X, D1225X, L1226X, K1227X, P1228X, V1229X, S1230X, E1231X, E1232X, V1233X, V1234X, E1235X, N1236X, P1237X, T1238X, I1239X, Q1240X, K1241X, D1242X, V1243X, L1244X, E1245X, C1246X, N1247X, V1248X, K1249X, T1250X, T1251X, E1252X, V1253X, V1254X, G1255X, D1256X, I1257X, I1258X, L1259X, M1376X, P1377X, T1378X, T1379X, I1380X, A1381X, K1382X, N1383X, T1384X, V1385X, K1386X, S1387X, V1388X, G1389X, K1390X, F1391X, C1392X, L1393X, E1394X, A1395X, S1396X, F1397X, N1398X, Y1399X, L1400X, K1401X, S1402X, P1403X, N1404X, F1405X, S1406X, K1407X, L1408X, I1409X, N1410X, I1411X, I1412X, I1413X, W1414X, F1415X, L1416X, L1417X, L1418X, S1419X, V1420X, C1421X, L1422X, G1423X, S1424X, L1425X, I1426X, Y1427X, S1428X, T1429X, A1430X, A1431X, L1432X, G1433X, V1434X, L1435X, M1436X, S1437X, N1438X, L1439X, G1440X, M1441X, P1442X, S1443X, Y1444X, C1445X, T1446X, G1447X, T1456I
		NSP4 hCoV-19/Wuhan/WIV04/2019	100%	100%	0	no aa changes
		NSP5 hCoV-19/Wuhan/WIV04/2019	100%	100%	0	no aa changes
		NSP6 hCoV-19/Wuhan/WIV04/2019	100%	100%	0	no aa changes
		NSP7 hCoV-19/Wuhan/WIV04/2019	100%	100%	0	no aa changes
		NSP8 hCoV-19/Wuhan/WIV04/2019	100%	100%	0	no aa changes
		NSP9 hCoV-19/Wuhan/WIV04/2019	100%	100%	0	no aa changes
		NSP10 hCoV-19/Wuhan/WIV04/2019	100%	100%	0	no aa changes
		NSP11 hCoV-19/Wuhan/WIV04/2019	100%	100%	0	no aa changes
		NSP12 hCoV-19/Wuhan/WIV04/2019	99.9%	99.0%	1	P323L[#o]

		NSP13 hCoV-19/Wuhan/WIV04/2019	100%	100%	0	no aa changes
		NSP14 hCoV-19/Wuhan/WIV04/2019	100%	100%	0	no aa changes
		NSP15 hCoV-19/Wuhan/WIV04/2019	100%	100%	0	no aa changes
		NSP16 hCoV-19/Wuhan/WIV04/2019	100%	100%	0	no aa changes
		Spike hCoV-19/Wuhan/WIV04/2019	99.8%	100%	3	**V308L**, **P521R**[$#o], **D614G**[$#lo]
		NS3 hCoV-19/Wuhan/WIV04/2019	99.6%	100%	1	**Q57H**[#o]
		E hCoV-19/Wuhan/WIV04/2019	100%	100%	0	no aa changes
		M hCoV-19/Wuhan/WIV04/2019	100%	100%	0	no aa changes
		NS6 hCoV-19/Wuhan/WIV04/2019	100%	100%	0	no aa changes
		NS7a hCoV-19/Wuhan/WIV04/2019	100%	100%	0	no aa changes
		NS7b hCoV-19/Wuhan/WIV04/2019	100%	100%	0	no aa changes
		NS8 hCoV-19/Wuhan/WIV04/2019	100%	100%	0	no aa changes
		N hCoV-19/Wuhan/WIV04/2019	99.8%	100%	1	**R209I**

P18_rad004/ARTIC/medaka MN908947.3	GH	NSP1 hCoV-19/Wuhan/WIV04/2019	100%	100%	0	no aa changes
		NSP2 hCoV-19/Wuhan/WIV04/2019	99.8%	100%	1	**T85I**
		NSP3 hCoV-19/Wuhan/WIV04/2019	100%	100%	0	no aa changes
		NSP4 hCoV-19/Wuhan/WIV04/2019	100%	100%	0	no aa changes
		NSP5 hCoV-19/Wuhan/WIV04/2019	100%	100%	0	no aa changes
		NSP6 hCoV-19/Wuhan/WIV04/2019	100%	100%	0	no aa changes
		NSP7 hCoV-19/Wuhan/WIV04/2019	100%	100%	0	no aa changes
		NSP8 hCoV-19/Wuhan/WIV04/2019	100%	100%	0	no aa changes
		NSP9 hCoV-19/Wuhan/WIV04/2019	100%	100%	0	no aa changes
		NSP10 hCoV-19/Wuhan/WIV04/2019	100%	100%	0	no aa changes
		NSP11 hCoV-19/Wuhan/WIV04/2019	100%	100%	0	no aa changes
		NSP12 hCoV-19/Wuhan/WIV04/2019	99.9%	99.0%	1	**P323L**[#o]
		NSP13 hCoV-19/Wuhan/WIV04/2019	99.8%	100%	1	**V209I**
		NSP14 hCoV-19/Wuhan/WIV04/2019	100%	100%	0	no aa changes
		NSP15 hCoV-19/Wuhan/WIV04/2019	99.7%	100%	1	**D212V**
		NSP16 hCoV-19/Wuhan/WIV04/2019	100%	100%	0	no aa changes
		Spike hCoV-19/Wuhan/WIV04/2019	99.9%	100%	1	**D614G**[$#lo]
		NS3 hCoV-19/Wuhan/WIV04/2019	99.6%	100%	1	**Q57H**[#o]
		E hCoV-19/Wuhan/WIV04/2019	100%	100%	0	no aa changes
		M hCoV-19/Wuhan/WIV04/2019	100%	100%	0	no aa changes
		NS6 hCoV-19/Wuhan/WIV04/2019	100%	100%	0	no aa changes
		NS7a hCoV-19/Wuhan/WIV04/2019	100%	100%	0	no aa changes
		NS7b hCoV-19/Wuhan/WIV04/2019	100%	100%	0	no aa changes
		NS8 hCoV-19/Wuhan/WIV04/2019	100%	100%	0	no aa changes
		N hCoV-19/Wuhan/WIV04/2019	100%	100%	0	no aa changes

Right-click here to save/download **query summary report** table for archiving or import to Excel (Tab-separated, one query per line) (protseqs)
Right-click here to save/download **location to mutation summary report** table for archiving or import to Excel (Comma-separated)

Bioinformatics Institute
BII

Developed by A*STAR Bioinformatics Institute (BII), Singapore.
© 2021 A*STAR Bioinformatics Institute (BII).

CoVsurver
